# Elucidation of the mechanism by which manganese-iron Prussian blue nanozymes alleviate ischemic stroke damage in a mouse model

**DOI:** 10.4103/NRR.NRR-D-24-00837

**Published:** 2025-02-24

**Authors:** Xue Li, Chengyun Hu, Shanshan Luo, Yanhong Zhang, Bilu Li, Chao Wu, Zhengyan Wu, Jia Zhang, Chaoliang Tang

**Affiliations:** 1 Department of Anesthesiology, The First Affiliated Hospital of University of Science and Technology of China, Division of Life Sciences and Medicine, University of Science and Technology of China, Hefei, Anhui Province, China; 2 Department of Anesthesiology, Anhui Provincial Cancer Hospital, Hefei, Anhui Province, China; 3 Institute of Intelligent Machines, Hefei Institutes of Physical Science, Chinese Academy of Sciences, Hefei, Anhui Province, China

**Keywords:** BV-2 cells, gut microbiota, inflammatory response, ischemic stroke, manganese-iron Prussian blue nanozymes, neurological function, nuclear factor-κB, oxidative stress, short-chain fatty acids, Toll-like receptor 4

## Abstract

Ischemic stroke represents a significant global health challenge, frequently associated with intricate pathophysiological alterations. During ischemic stroke, the generation of reactive oxygen species markedly increases, leading to direct neuronal damage as well as initiating a cascade of inflammatory responses. This oxidative stress can also disturb the equilibrium of the gut microbiota, resulting in dysbiosis. In turn, an imbalance in gut microbiota can further exacerbate the production of reactive oxygen species and contribute to a pro-inflammatory environment within the body. This creates a vicious cycle that not only promotes the progression of stroke but also leads to adverse functional outcomes. The neuroinflammation and intestinal microbiota dysbiosis that occur following ischemic stroke are critical contributors to stroke progression and adverse functional outcomes. We previously developed manganese-iron Prussian blue nanozymes, characterized by a multi-enzyme structure and a porous design, that exhibit strong antioxidant properties. However, the therapeutic effects of manganese-iron Prussian blue nanozymes on ischemic stroke and their mechanisms of action remain have not been fully elucidated. To investigate this, we constructed a mouse model of middle cerebral artery occlusion and administered manganese-iron Prussian blue nanozymes via gastric gavage. Our results demonstrated that these nanozymes substantially reduced infarct volume, improved neurological function, restored gut microbiota balance, and increased levels of short-chain fatty acids in the mouse model. Treatment of lipopolysaccharide-treated BV-2 cells with short-chain fatty acids markedly decreased the expression levels of components of the Toll-like receptor 4/nuclear factor kappa B signaling pathway, including Toll-like receptor 4, inhibitor of nuclear factor kappa-B kinase subunit alpha, and pp65. These findings suggest that manganese-iron Prussian blue nanozymes can correct gut microbiota dysbiosis and increase short-chain fatty acid production by modulating the Toll-like receptor 4/nuclear factor kappa B signaling pathway, thereby providing therapeutic benefits in the context of ischemic stroke.

## Graphical abstract

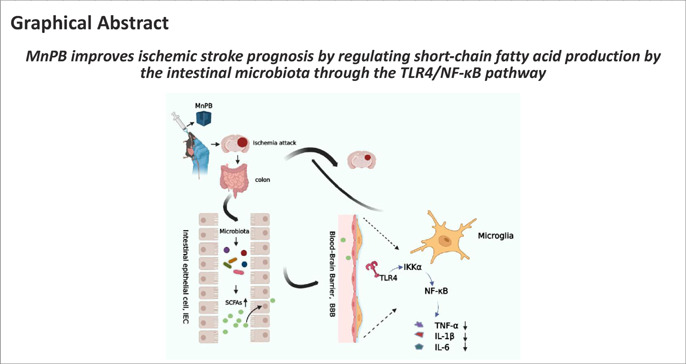

## Introduction

Ischemic stroke (IS) is the predominant form of stroke globally and is a major cause of disability and mortality (Tang et al., 2020; Lv et al., 2022; Dai et al., 2024). The pathophysiological processes of IS are highly complex and involve multiple interrelated mechanisms. Among these, the over -production of reactive oxygen species (ROS) during ischemia and subsequent reperfusion is a key event (Huang et al., 2025). ROS, including hydrogen peroxide, hydroxyl radicals, and superoxide anions, can directly damage cellular components such as lipids, proteins, and DNA, leading to neuronal cell death and exacerbating brain tissue injury (Chen et al., 2019c). Simultaneously, this oxidative stress also disrupts the normal composition and function of the gut microbiota. Current therapeutic strategies for IS, such as pharmacological thrombolysis and mechanical thrombectomy performed within a critical time window (Jia et al., 2024), are crucial but not without risks because cerebral ischemia-reperfusion injury is a common complication (Kong et al., 2024). Hence, the pursuit of novel therapeutic approaches for IS is imperative (Tang, 2024).

In recent years, the interplay between the gut microbiota and human health has garnered significant interest. The gut microbiota, which plays a crucial role in maintaining overall health, forms a bidirectional communication pathway with the brain known as the microbiota–gut–brain axis (Cryan et al., 2019; Cheng et al., 2024; Liu et al., 2024). Accumulating evidence underscores the significance of gut microbiota dysregulation and oxidative stress in IS prognosis (Zhao et al., 2018b; Wu et al., 2024). We previously showed that manganese-iron Prussian blue (MnPB) nanozymes possess remarkable antioxidant capabilities, effectively neutralizing ROS within the body, thereby mitigating inflammation and cellular damage (Hu et al., 2024). A recent study found that IS rapidly causes intestinal ischemia and the production of excessive nitrate through free radical reactions, leading to disordered expansion of the intestinal flora (Xu et al., 2021). In view of the similar pathophysiological mechanisms of intestinal changes caused by inflammatory bowel disease and IS, such as oxidative stress and inflammatory injury, we hypothesized that MnPB could also help diagnose and treat IS. Short-chain fatty acids (SCFAs) produced by the gut microbiota are implicated in the pathophysiology of central nervous system disorders (Dalile et al., 2019) and serve as pivotal signaling molecules for gut-brain communication (O’Mahony et al., 2015). Notably, a negative correlation exists between IS and SCFA levels, with butyric acid showing the strongest decrease in response to IS. Administration of butyric acid to rats with cerebral ischemia has been shown to significantly reduce neurological injury and infarct volume, as well as alleviate cerebral edema and lower serum cholesterol, triglycerides, fibrinogen levels, blood viscosity, and intestinal permeability (Chen et al., 2019c). Moreover, SCFAs are believed to cross the blood-brain barrier and exert neuromodulatory effects (Mitchell et al., 2011), as evidenced by the presence of SCFA receptors, such as free fatty acid receptor 3, on blood–brain barrier endothelial cells. These receptors, upon lipopolysaccharide stimulation, interact with Toll-like receptor 4 (TLR4) in the context of CD14 and LY96 (MD2) to initiate inflammatory responses (Hoyles et al., 2018). Upon activation, TLR4, which is widely expressed in the central nervous system, including by microglia, neurons, astrocytes, and endothelial cells, triggers the nuclear factor kappa-B (NF-κB) pathway, leading to the production of pro-inflammatory cytokines such as interleukin-6 (IL-6), interleukin-1β (IL-1β), and tumor necrosis factor α (TNF-α) (Wang et al., 2021). BV-2 cells, the resident phagocytic immune cell in the brain, are easily activated by excessive production of ROS. Although BV-2 activation has beneficial effects on cell maintenance and innate immunity, continued activation results in the production of a number of cytotoxic mediators. Activated BV-2 cells induce tissue damage by releasing chemokines and inflammatory factors (such as TNF-α, IL-1β, and IL-6), and finally lead to apoptosis through a series of pathways (Ma et al., 2022; Liu et al., 2023). Therefore, we decided to use BV-2 cells as an *in vitro* model to investigate the effects of MnPB nanozymes. The aim of this study was to delineate the role of MnPB nanozymes in a mouse model of IS and explore the underlying mechanisms, potentially offering insight into therapeutic options for IS patients.

## Methods

### Animals and *in vivo* experimental design

Adult specific pathogen free male C57BL/6J mice (*n* = 24, aged 6–7 weeks and weighing 22–26 g) were sourced from Hefei Gue Kangyuan Innovative Medicine Research Co., Ltd (Heifei, Anhui, China; license No. SCXK (Zhe) 2019-0001). Adult male germ-free (GF) and wild type (WT) C57BL/6J mice (*n* = 16, aged 6–7 weeks and weighing about 25 g, were sourced from GemPharmatech Co., Ltd. (Nanjing, Jiangsu, China, license No. SCXK (Su) 2023-0009). The animals were maintained under specific pathogen-free conditions with a 12/12-hour light/dark cycle at a constant temperature of 25°C. The Animal Ethics Committee of the University of Science and Technology of China granted approval for all experimental procedures (approval date: February 16, 2023; approval No. 2023-N(A)-011). All experiments were designed and reported according to the Animal Research: Reporting of *In Vivo* Experiments (ARRIVE) guidelines (Percie du Sert et al., 2020).

Following a 1-week acclimatization period to the environment, the C57BL/6J mice were randomly assigned to one of three groups, each consisting of eight animals: the Control group, which underwent a sham operation; the middle cerebral artery occlusion (MCAO) + normal saline (NS) group; and the MCAO + MnPB group. Commencing 24 hours post-MCAO/sham surgery, all groups received gavage feeding of either 2.5 mg/mL MnPB nanozymes (10 mg/kg) or an equivalent volume of NS on days 2, 4, and 6. Body weight was recorded daily. In addition, WT and GF mice (*n* = 8 each) were subjected to MCAO and treated with 10 mg/kg MnPB nanozymes (2.5 mg/mL) as described above 24 hours later. MCAO was performed using a well-established method, and the surgery was performed on the right side of the mouse brain (Zhang et al., 2019). Throughout the surgical procedure, the ambient room temperature was strictly regulated between 24°C and 25°C. Neurological severity scoring was conducted using the Zea-Longa scale 24 hours post-surgery (Li et al., 2023), with a higher score indicating greater behavioral impairment, reflecting more severe neurological deficit. Mice that scored between 1 and 3 were selected for subsequent analysis. The experimental design is shown in **Additional Figure 1**, and the number of experimental animals used in the *in vivo* experiments is shown in **Additional Table 1**.

**Additional Table 1:** Number of experimental animals in the *in vivo* experiments

Experimental method	Number of animals/samples each group
Morris water maze	5
TTC staining	6-8
HE staining	6
TUNEL staining	6
ELISA	5
Western blotting	3
16S rRNA	5

16S rRNA: 16S ribosomal RNA; ELISA: enzyme-linked immunosorbent assay; HE: hematoxylin and eosin; TTC: 2,3,5-Triphenyltetrazolium chloride; TUNEL: terminal deoxynucleotidyl transferase dUTP nick-end labeling.

### Preparation and characterization of MnPB nanozymes

The MnPB nanozymes were prepared as described in detail in our previous study (Hu et al., 2024). MnPB morphology and microstructure were characterized by field emission scanning electron microscopy (HD-2700, Hitachi Co., Hitachi, Japan) and transmission electron microscopy (JEM-F200, JEOL Ltd., Tokyo, Japan).

### Morris water maze test

Following the surgical procedure, the mice were subjected to the Morris water maze test over a period of 6 consecutive days to assess their spatial memory (Kong et al., 2024). The Morris water maze apparatus (XR-XM101, Shanghai Xinruan Information Technology Co., Ltd, Shanghai, China) consisted of a circular black pool with a diameter of 150 cm and a height of 50 cm, filled to a depth of 25 cm with water maintained at a temperature of 20 ± 1°C. The pool was conceptually divided into four equal quadrants labeled 1–4, with a circular escape platform positioned in quadrant 1, submerged 2 cm below the water surface. The mice underwent two trials daily for the initial 5 days, each lasting for 60 seconds. After each trial, the mice were permitted to rest on the platform for 10 seconds. On the 6^th^ day, a probe trial was conducted by removing the platform, allowing the mice to navigate the pool freely for 60 seconds. The time taken by the mice to complete the task and the path traversed were meticulously recorded using video tracking. The collected data collected were then analyzed using specialized ANY-maze software (Version 7.0, Stoelting Co., Ltd, Wood Dale, IL, USA), ensuring objective and quantitative assessment of mouse spatial memory.

### 2,3,5-Triphenyltetrazolium chloride staining for cerebral infarction assessment

The mice were anesthetized via inhalation of isoflurane (5%, Ruiwode, Shenzhen, Guangdong, China). Cardiac perfusion was performed. The brain tissue was collected and then frozen immediately at –20°C for 20–30 minutes (Sun et al., 2018). Subsequently, the tissue was sectioned into continuous 2-mm-thick coronal slices. The slices were then immersed in a 2% solution of 2,3,5-triphenyltetrazolium chloride (TTC; T8877, Sigma-Aldrich, St. Louis, MO, USA) for staining. The extent of cerebral infarction in each section was quantified using ImageJ software (Version 1.9.0, National Institutes of Health, Bethesda, MD, USA; Schneider et al., 2012). The cerebral infarction volume was calculated as follows: cerebral infarction volume (%) = (sum of the volume of white ischemic area in each section/sum of the volume of brain slices in each section) × 100.

### Histological assessment

Mouse brain, colon, and other tissues were harvested and fixed in a 4% paraformaldehyde solution for 24 hours. Following fixation, the tissues were dehydrated and embedded in paraffin. Using a microtome, the embedded tissues were carefully cross-sectioned into 5-μm-thick slices. The sections were then stained with hematoxylin and eosin (HE). The stained sections were examined under TissueFAXS Plus S (TissueGnostics, Vienna, Austria) to assess histological changes.

### Enzyme-linked immunosorbent assay

To quantify the levels of TNF-α, IL-1β, and IL-6 in both brain and colon tissues, enzyme-linked immunosorbent assay kits from Elabscience (Wuhan, Hubei, China) were used according to the manufacturer’s protocols. Absorbance readings were obtained using a spectrophotometer from Molecular Devices (San Jose, CA, USA) and converted into concentrations, which were expressed in picograms per milliliter (pg/mL), providing a quantitative assessment of the cytokine levels in the samples (Tang et al., 2021).

### Terminal deoxynucleotidyl transferase dUTP nick-end labeling staining

Mouse brains were harvested and fixed in a 4% paraformaldehyde solution for 24 hours. Following fixation, the tissues were dehydrated and embedded in paraffin. Using a microtome (RM2235, Leica, Weztlar, Germany), the embedded tissues were carefully cross-sectioned into 5-μm-thick slices. This was followed by staining using a terminal deoxynucleotidyl transferase dUTP nick-end labeling (TUNEL) fluorescent apoptosis kit (Vazyme, Nanjing, Jiangsu, China). The stained sections were examined using TissueFAXS Plus S (TissueGnostics) to assess histological changes.

### Cell culture and *in vitro* experimental design

The human normal colonic epithelial cell line NCM460 (American Type Culture Collection, Manassas, VA, USA, CRL-1807, RRID:CVCL_2872) and BV-2 microglial cells (American Type Culture Collection, CRL-2467, RRID:CVCL_5744) were cultured in Dulbecco’s modified Eagle’s medium (Gibco, Carlsbad, CA, USA, Cat# 11965092) containing 10% fetal bovine serum (Thermo Fisher Scientific, Waltham, MA, USA, Cat# A5669701) and 1% of an antibiotic mixture containing penicillin/streptomycin (Gibco, Cat# 15140122). The cells were maintained in a humidified incubator at 37°C with a 5% CO_2_ atmosphere. The NCM460 cells were subcultured every 3–4 days, while BV-2 cells were passaged every 1–2 days. All experimental procedures were executed during the logarithmic (exponential) phase of cell growth to ensure optimal cellular activity.

To assess the cytotoxicity of MnPB, NCM460 cells were seeded into 96-well plates at a density of 1 × 10^4^ cells per well and then exposed to different concentrations of MnPB (0, 6.25, 12.5, 25, 50, or 100 μg/mL) for 12 hours. To evaluate cellular uptake of MnPB, rhodamine B isothiocyanate (RBITC; GLPBIO, Montclair, CA, USA, CAT# GC19883) was mixed with an equal concentration of MnPB solution for 2 hours in the dark to fluorescently label the nanomaterials. Then, NCM460 cells were seeded at a density of 1 × 10^4^ cells per well in 12-well plates and exposed to different concentrations of the fluorescent nanomaterials (0, 6.25, 12.5, 25, or 50 μg/mL). After 4 hours of treatment, the cells were fixed with paraformaldehyde, and the nuclei were stained with 4′,6-diamidino-2-phenylindole (GLPBIO, Cat#GC43378) and imaged using a fluorescence microscope (LSM 980, Carl Zeiss AG, Oberkogen, Germany).

To assess the cytotoxicity of SCFAs, BV-2 cells were seeded into 96-well plates at a density of 6 × 10^3^ cells per well. Then, they were exposed to different concentrations of sodium acetate (NaA; Macklin, Shanghai, China, Cat#127-09-3) (0, 20, 40, 60, 80, or 100 mM), sodium propionate (NaP; Macklin, Cat#137-40-6) (0, 1, 2, 4, 8, or 16 mM), and sodium butyrate (NaB; Macklin, Cat#156-54-7) (0, 0.1, 0.2, 0.4, 0.8, 1.6, or 3.2 mM) for 12 hours. The results showed that treating BV-2 cells for 12 hours with NaA 20 mM, NaP 1 mM, and NaB 1 mM did not affect cell viability.

To simulate oxidative stress injury *in vitro*, BV-2 cells were seeded into 96-well plates at a density of 6 × 10^3^ cells per well and exposed to different concentrations of H_2_O_2_ (0, 0.25, 0.5, 0.75, and 1 mM) (Sinopharm Chemical Reagent Co., Ltd, Shanghai, China, Cat# 10011218) for 4 hours. On the basis of the results, 1 mM was selected as the optimal concentration; in future experiments, cells were preincubated with SCFAs with or without MnPB, followed by exposure to 1 mM H_2_O_2_ for 4 hours. To evaluate MnPB cytotoxicity, BV-2 cells were seeded into 96-well plates at a density of 6 × 10^3^ cells per well and exposed to different concentrations of MnPB (0, 6.25, 12.5, 25, 50, 100, or 200 μg/mL). Cell viability was determined by cell counting kit-8 (CCK-8, Vazyme): 10% CCK-8 solution to each well according to the manufacturer’s instructions, and the plates were incubated at 37°C with 5% CO_2_ for 2.5 hours. Finally, cell viability was determined by measuring the absorbance at 450 nm using a spectrophotometer (Molecular Devices).

Intracellular ROS levels were measured using the fluorescent probe 2,7-dichlorofluorescein diacetate (Beyotime Biotechnology, Shanghai, China). Apoptosis rates were determined using an apoptosis kit (Elabscience). Both ROS levels and apoptosis rates were assessed via flow cytometry using a BD FACSVerse (Beckman Coulter, Brea, CA, USA) instrument.

### Western blotting

Brain tissue samples were homogenized to yield a uniform mixture and centrifuged, and the supernatant was collected. For BV-2 cells, protein lysates were prepared using radio immunoprecipitation assay lysis buffer. The protein concentrations in the samples were determined using a bicinchoninic acid protein assay kit, and samples were normalized to a known amount of bovine serum albumin before being loaded onto the gel, maintaining a consistent protein loading amount of 30–40 μg per lane. Proteins were separated using 7.5% or 10% sodium dodecyl sulfate-polyacrylamide gel electrophoresis and transferred to polyvinylidene fluoride membranes. The membranes were blocked in 5% non-fat milk and then incubated with the following primary antibodies overnight at 4°C: TLR4 rabbit mAb (1:1000, Servicebio, Wuhan, Hubei, China, Cat# GB11519, RRID: AB_3186234), inhibitory kappa B kinase α (IKKα) rabbit mAb (1:1000, Zenbio, Chengdu, Sichuan, China, Cat# R24674, RRID: AB_3665162), and phosphorylated nuclear factor kappa-B p65 (P-NF-κB p65 (pp65)) rabbit mAb (1:1000, Cell Signaling, Danvers, MA, USA, Cat# 3033, RRID: AB_331284). After incubation with primary antibodies, the membranes were further incubated with the corresponding horseradish peroxidase-conjugated secondary antibody (goat anti rabbit IgG HRP conjugated, 1:3000, Servicebio, Cat# GB23303, RRID: 2811189) for 2 hours at room temperature (20–25°C). The blots were developed using an enhanced chemiluminescence kit, and band detection and quantification were performed using ImageJ software. All results were expressed as relative ratios to the signal from the corresponding anti-glyceraldehyde 3-phosphate dehydrogenase (GAPDH) rabbit mAb (1:1000, Abcam, Cambridge, UK, Cat# ab8245, RRID: AB_2107448), which served as an internal control for protein loading.

### Gut microbiota sequencing analysis

Fresh fecal samples were collected aseptically from the colons of five mice per group, as samples from the colon are ideal for the determination of intestinal flora (Tan et al., 2024). Stool samples were collected in the morning to avoid changes in microbial composition at different times of the day. The feces were collected in a well-lit and ventilated room. Feces were stored immediately after collection in sterile tubes and immersed in liquid nitrogen to maintain the integrity of the microbiota. Extraction of total DNA from the fecal samples and analysis of microbial composition through 16S ribosomal RNA gene sequencing were carried out by Biomarker Technologies (Shandong, China). The V3–V4 hypervariable region of the bacterial 16S ribosomal RNA gene, known for its discriminatory power in microbial identification, was targeted for amplification by polymerase chain reaction. The polymerase chain reaction was performed using a specific primer pair designed to anneal to conserved regions flanking the V3–V4 region, with the following sequences: forward primer, 5′-ACT CCT ACG GGA GGC AGC A-3′, and reverse primer, 5′-GGA CTA CHV GGG TWT CTA AT-3′. Data analysis was performed using PICRUSt2 software and the Kyoto Encyclopedia of Genes and Genomes (KEGG) database, provided by BMKCloud (www.biocloud.net).

### Short-chain fatty acid analysis

At the end of the experiment, we collected fresh colon feces and brain tissue from the mice in each group under sterile conditions. Fresh fecal samples were collected and promptly snap-frozen in liquid nitrogen for over 15 minutes to ensure rapid preservation of microbial metabolites. These samples were then stored at –80°C to maintain their integrity until analysis. Concurrently, the targeted brain tissue was carefully excised, rinsed in phosphate buffer saline to eliminate blood and debris, and subsequently dissected into smaller fragments. The tissue fragments were flash-frozen by quickly immersing them in liquid nitrogen using forceps. Following these preparatory steps, the samples were shipped to Sangon Biotech (Shanghai, China), a specialized service provider, for quantitative analysis of SCFAs. This procedure ensures that the SCFAs are accurately measured and identified, providing valuable insights into the metabolic activity of the gut microbiota and its potential influence on neurological health.

### Statistical analysis

G*Power 3.1.9.2 software (Heinrich-Heine-Universität Düsseldorf, Düsseldorf, Germany) was used to determine the sample size for the mouse experiments. The investigators who performed the statistical analysis were blinded to the group assignments. Data are presented as the mean ± standard deviation (SD). Unpaired Student’s *t*-test was used to detect statistically significant differences between two groups, and one-way analysis of variance followed by a *post hoc* Tukey test was used to compare multiple groups. This approach allows for the identification of specific group differences while controlling the overall type I error rate. To elucidate the relationships between various parameters, Pearson’s correlation analysis was used. This statistical method is effective for assessing the strength and direction of linear associations between continuous variables. We performed the statistical analyses using GraphPad Prism 9.5.1 statistical software (GraphPad Software, Boston, MA, USA, www.graphpad.com) and considered *P* values below 0.05 to indicate statistical significance.

## Results

### Characterization and biosafety of MnPB nanozymes

**Figure 1:**
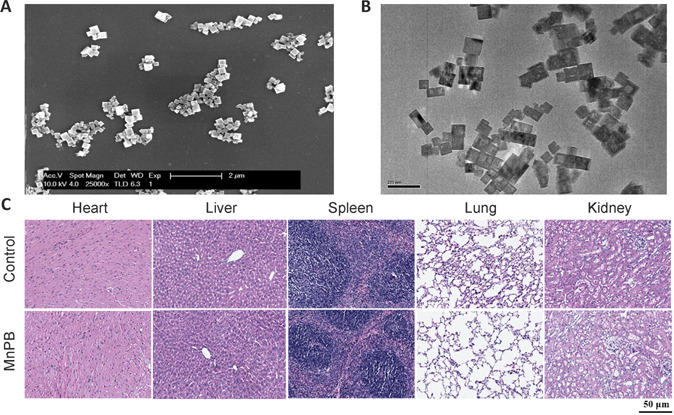
Structural and chemical characterizations of the MnPB nanozymes. (A) Scanning electron microscopy images reveal the surface morphology of the nanoparticles. MnPB was near cubic-shaped crystallites. Scale bar: 2 μm. (B) Transmission electron microscopy images depict the internal structure and confirm the crystalline nature of the MnPB nanozymes. MnPB was near cubic-shaped crystallites. Scale bar: 200 nm. (C) HE stained sections of heart, liver, spleen, lungs, and kidneys from mice in both the Control and MnPB-treated groups show the histological assessment of tissue integrity and the absence of overt toxicity. Scale bar: 50 μm. HE: Hematoxylin and eosin; MnPB: manganese-iron Prussian blue.

To explore the potential of MnPB nanozymes as a therapeutic agent, it is essential to first characterize their structure and assess their biosafety. MnPB nanozymes were synthesized by combining 0.2 mmol of Mn^2+^ ions with 0.5 mmol of potassium ferricyanide (K_4_Fe(CN)_6_) in a citric acid solution at 60°C. The resulting multifunctional MnPB nanoparticles were small and exhibited a simplified interlayer structure, as previously described (Hu et al., 2024). The morphology of the nanozymes was characterized using high-resolution scanning electron microscopy (**Figure 1A**) and transmission electron microscopy (**Figure 1B**), revealing a subcubic crystalline structure. To assess the *in vivo* biosafety of the MnPB nanozymes, mice in the control and experimental groups were subjected to oral gavage with equal volumes of NS and 10 mg/kg MnPB nanozymes, respectively. This dose was based on our previous study (Hu et al., 2024). The mice in the experimental group were treated with MnPB nanozymes 24 hours after MCAO, while the mice in the control group were treated with the same volume of normal saline 24 hours after the sham operation. All groups received oral gavage of either NS or MnPB nanozymes on days 2, 4 and 6, and mice were sacrificed on day 8. After euthanizing the mice, the heart, liver, spleen, lung, and kidney were collected, fixed in paraformaldehyde, and stained with HE (Hu et al., 2024). Histological examination showed no significant damage or structural alterations in the organs between the control and MnPB-treated groups (**Figure 1C**), indicating the high biosafety profile of the MnPB nanozymes. In conclusion, MnPB nanozymes have a specific crystal structure and exhibit high biosafety, which lays the foundation for further exploration of their therapeutic effects.

### MnPB nanozymes alleviate infarction symptoms and modulate behavioral changes in mice subjected to middle cerebral artery occlusion

**Figure 2:**
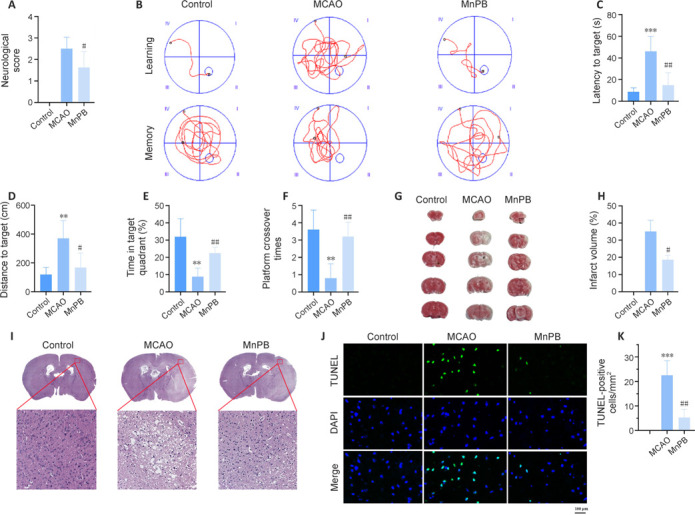
MnPB nanozymes improve the neurobehavioral outcomes and infarct size in MCAO mice. (A) Neurobehavioral scores (*n* = 8). Therapeutic effect of MnPB nanozymes on post-ischemic behavioral deficits. (B) Representative swimming tracks from the Morris water maze test demonstrate the spatial learning and memory capabilities of mice (*n* = 5). (C, D) The latency to reach the target (C) and the distance traveled to the target (D), respectively, in the hidden platform test for mice following different treatments (*n* = 5). The escape latency and the distance to reach the target decreased significantly in the MCAO + MnPB group. (E, F) The time spent in the target quadrant (E) and the number of platform crossovers (F), reflect spatial memory performance after treatment (*n* = 5). (G) Representative images of TTC-stained brain sections from embolic MCAO mice on day 7, visualizing the infarct area (white) (*n* = 3). MnPB nanozyme treatment reduced infarct volume compared with MCAO mice. (H) Quantification of the infarct volume as a percentage of the total brain volume on day 7, illustrates the neuroprotective effect of MnPB nanozymes (*n* = 3). (I) HE staining images of ischemic penumbral sections of mice after MCAO, with obvious nuclear condensation and inflammatory cell infiltration, which was alleviated by MnPB nanozyme inhibition (*n* = 3). Scale bar: 50 μm. (J) Representative image of TUNEL staining (green) in ischemic penumbra area, nucleus with DAPI (blue). MnPB treatment decreased the number of TUNEL-positive cells. Scale bar: 100 μm. (K) Quantitative analysis of TUNEL-positive cells (*n* = 3). Data are expressed as mean ± SD. **P < 0.01, ***P < 0.001, *vs*. Control group; #*P* < 0.05, ##*P* < 0.01, *vs*. MCAO group (one-way analysis of variance followed by Tukey’s *post hoc* test). DAPI: 4′,6-Diamidino-2-phenylindole; HE: hematoxylin and eosin; MCAO: middle cerebral artery occlusion; MnPB: manganese-iron Prussian blue; TUNEL: terminal deoxynucleotidyl transferase dUTP nick-end labeling.

The core aim of this research was to determine whether MnPB nanozymes could alleviate the adverse effects of MCAO in mice, especially in terms of infarction symptoms and behavioral alterations. Following surgery, mice received equivalent volumes of NS and 10 mg/kg MnPB nanozymes. At the end of the treatment period, neurobehavioral assessments were conducted, complemented by TTC staining of brain tissue and histological examination with HE staining. The day after MCAO, behavioral patterns across all groups were evaluated using the Zea-Longa scoring system. As shown in **Figure 2A**, the Control group scored 0, while the MCAO + NS group had a score of 2.5 ± 0.53. Notably, the MCAO + MnPB group showed a significantly reduced score of 1.625 ± 0.74, indicating substantial improvement compared with the MCAO + NS group (*P* < 0.05). These results suggest that MnPB nanozymes have significant potential to mitigate the neural dysfunction caused by ischemia and reperfusion in mice subjected to MCAO. To further assess the therapeutic effects of MnPB nanozymes on IS, cognitive function was evaluated using the Morris water maze test. The recovery rate in MCAO mice was quantified, and the swimming trajectories in **Figure 2B** illustrate the learning capacity of the mice. Post-IS, there was an increase in escape latency and the distance traveled to the target location (**Figure 2C** and **D**). However, MnPB nanozyme treatment significantly reduced these parameters to levels nearly identical to those of the control group, indicating a restorative effect on cognitive function. Next, spatial memory was evaluated by removing the visible platform, and the time spent in the target sector and the frequency of platform zone crossings were recorded (**Figure 2E** and **F**). Post-ischemia, a decrease in time spent in the target quadrant and fewer platform area crossings were observed, indicating impaired spatial memory. MnPB nanozyme treatment, however, led to significant restoration of spatial memory, as evidenced by increased time spent in the target quadrant and more frequent platform area crossings. To visually assess the therapeutic potency of MnPB nanozymes, we examined TTC-stained brain sections taken from MCAO mice on the eighth day (**Figure 2G**). The cerebral infarction volume showed a significant decrease from 35.12% ± 6.56% in the NS group to 18.68% ± 2.56% after MnPB nanozyme treatment (**Figure 2H**), demonstrating the treatment’s effectiveness in reducing infarct size. Furthermore, brain tissue sections were subjected to HE and TUNEL staining (**Figure 2I** and **J**). A reduction in cell volume and obvious nuclear atrophy were observed in the ischemic penumbra area in the NS group. By contrast, MnPB nanozyme treatment preserved normal cellular morphology to levels comparable to those seen in the normal group (**Figure 2I**). Pathological examination indicated that MnPB nanozymes provided robust neuroprotection. TUNEL staining was used to observe the effect of MnPB nanozymes on apoptosis in the ischemic penumbra area after MCAO. There were no apoptotic cells in the control group, while the number of TUNEL-positive cells was significantly increased in the MCAO model group. Notably, MnPB nanozyme treatment substantially suppressed the increase in TUNEL fluorescence signal seen in mice subjected to MCAO (**Figure 2J** and **K**). These findings confirm that MnPB nanozyme treatment significantly improves IS symptoms and promotes brain recovery in mice subjected to MCAO.

### MnPB nanozymes modulate gut microbiota dysbiosis in mice subjected to middle cerebral artery occlusion

**Figure 3:**
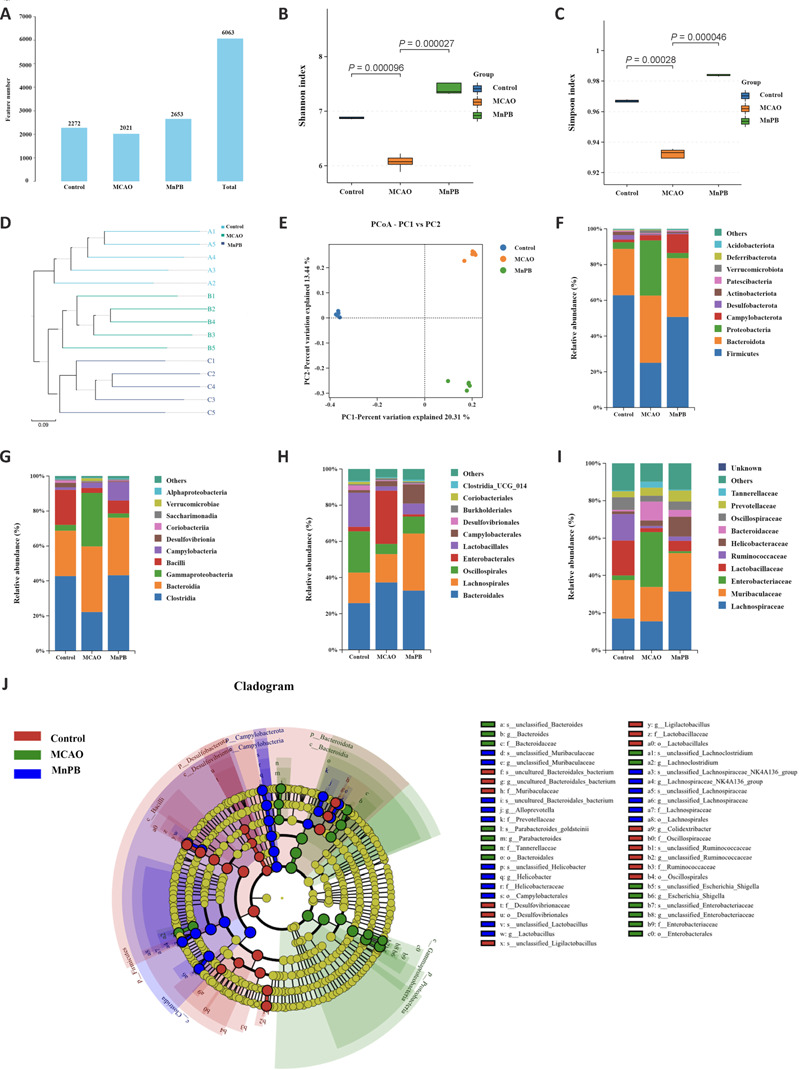
Effects of MnPB nanozymes on gut microbiota in MCAO model mice (*n* = 5). (A) Alpha diversity analysis of operational taxonomic units representing the fecal gut microbiota in mice, indicating the richness and evenness of microbial communities. (B) Shannon index, a measure of gut microbiota diversity, indicating the balance and variability of microbial species (one-way analysis of variance followed by Tukey’s *post hoc* test). (C) Simpson index, another diversity metric reflecting the concentration and dominance of species within the microbiota (one-way analysis of variance followed by Tukey’s *post hoc* test). (D) Principal coordinate analysis (PCoA) based on unweighted UniFrac distances, illustrating the beta diversity and distinguishing microbial community structures between samples. (E) Hierarchical clustering dendrogram of the sample grouping based on microbial composition, highlighting the differences between the Control, MCAO, and MnPB treatment groups. (F) Mean composition at the phylum level, revealing the major groups of gut microbes and their relative abundances across treatment conditions. (G) Order-level composition, providing insights into the specific orders within the gut microbiota that are affected by the MCAO model and MnPB nanoenzyme treatment. (H) Family-level composition, identifying the families of bacteria that exhibit significant differences in response to the treatments. (I) Comparative analysis of gut microbiota at the phylum, order, and family levels, demonstrating the shifts in microbial populations due to the MCAO model and the restorative effects of MnPB nanozymes. (J) Circular branching plot from the linear discriminant analysis effect size. The microbial taxa with significant differences in abundance between the groups. MCAO: Middle cerebral artery occlusion; MnPB: manganese-iron Prussian blue.

The gut microbiota is recognized for its significant role in IS pathogenesis, with evidence supporting a bidirectional gut-brain axis facilitated by microbial communication (Chidambaram et al., 2022). Thus, it was important to investigate how MnPB nanozymes influenced gut microbiota dysbiosis in MCAO mice. We conducted a comprehensive 16S ribosomal RNA gene sequencing analysis across variable regions V3–V4 in fecal samples from all experimental groups to evaluate alterations in the gut microbiota induced by MCAO and treatment with MnPB. We observed both common and unique operational taxonomic units among the groups (**Figure 3A**). Quantitative analysis revealed a reduction in gut microbiota diversity in the MCAO mice that was restored following treatment with MnPB nanozymes. Assessment of species diversity using the Shannon index (**Figure 3B**) and the Simpson index (**Figure 3C**) demonstrated that MnPB nanozymes enhanced the diversity of the gut microbiota. Principal coordinate analysis based on UniFrac distances highlighted distinct clustering patterns for each group, indicative of their unique microbiota compositions (**Figure 3D**). Hierarchical clustering further differentiated the microbial communities in the feces of the MCAO and MnPB groups from those of the Control group (**Figure 3E**). These findings suggest that MnPB nanozyme treatment may improve behavioral and cognitive deficits in MCAO mice by rectifying gut microbiota imbalances. Further analysis of the gut microbiota at the phylum level (**Figure 3F**) identified significant intergroup differences in the relative abundance of *Firmicutes*, *Bacteroidota*, *Proteobacteria*, and *Campylobacteria*. In MCAO model mice, an increase in *Bacteroidota* and *Proteobacteria* was observed, as well as a decrease in Firmicutes, and these effects were mitigated by MnPB nanozyme treatment. At the class level, we noted an increase in *Clostridia* and a decrease in Bacilli in the MCAO group, with *Bacteroidia* and *Gammaproteobacteria* showing elevated relative abundances. MnPB nanozymes effectively reversed these changes (**Figure 3G**). Order-level analysis revealed an increase in *Bacteroidales* and a decrease in *Lachnospirales* in the MCAO group, with MnPB nanozymes modulating these shifts (**Figure 3H**). At the family level, the relative abundance of *Lachnospiraceae* was decreased in the model group, which was ameliorated by MnPB nanozyme treatment. Additionally, there was a significant increase in *Enterobacteriaceae* abundance in the MCAO group that was attenuated following MnPB nanozyme treatment (**Figure 3I**). The results suggested that MnPB nanozymes could rectify gut microbiota imbalances, potentially contributing to the improvement of behavioral and cognitive deficits in MCAO mice.

### MnPB nanozymes enhance short-chain fatty acid content and modulate the gut microbiota

**Figure 4:**
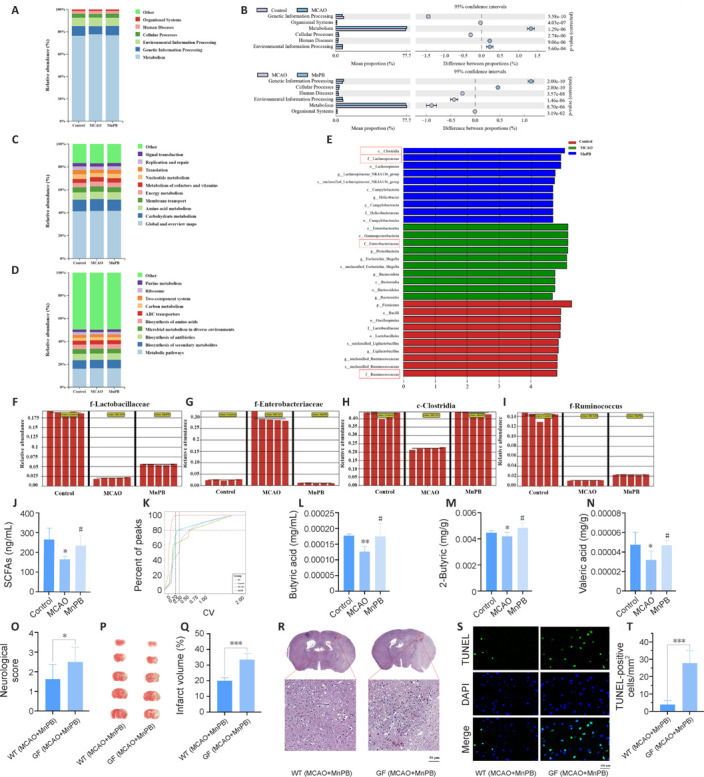
Gut microbiota metabolic function prediction in MCAO model mice. (A) Utilizing the PiCRUSt bioinformatics software package, the relative abundance of microbial gene functions among different mouse groups were present, indicating the predicted metabolic capabilities of the gut microbiota. (B) Differential analysis of KEGG metabolic pathways reveals distinctions in metabolic activity between the groups. (C) Metabolic functions at the second level of the hierarchy, providing a broad overview of the predominant metabolic processes. (D) Metabolic functions at the third level of the hierarchy, offering more granular insights into specific metabolic activities. (E) Linear discriminant analysis effect size analysis highlights significant differences in species abundance across the Control, MCAO, and MnPB groups, with a log LDA score threshold > 4.0 indicating discriminant features. (F–I) Quantification of specific bacterial taxa, including Lactobacillaceae, Clostridia, Ruminococcaceae, and Enterococcaceae, showing variations in their numbers among the different groups. (J) Measurement of SCFA contents. (K) Quality control assessment of the CV values for brain tissue mixed solutions, ensuring the stability and reliability of the experimental data. Over 80% of substances with CV values less than 0.3 indicate stable data, while CV values less than 0.2 in over 80% of substances denote very stable data. (L–N) The effects of MnPB nanozyme therapeutic treatments on SCFAs levels in brain tissue samples of MCAO mice are shown for butyric acid, 2-methylbutyric acid, and valeric acid, respectively (*n* = 3). (O) Neurobehavioral scores for the Control group, WT (MCAO + MnPB) group, and GF (MCAO + MnPB) group (*n* = 8), indicating the therapeutic effect of MnPB nanozymes on post-ischemic behavioral deficits. (P) Representative images of TTC-stained brain sections from embolic MCAO mice on day 8, visualizing the infarct area (white) (*n* = 4). (Q) Quantification of the infarct volume as a percentage of the total brain volume on day 8, illustrates the neuroprotective effect of MnPB nanozymes (*n* = 4). (R) Representative pathological examination images of brain slices on the 8^th^ day of embolization, with enlarged ischemic penumbra area, and HE staining to show the protective effect of MnPB nanozyme on brain tissue (*n* = 3). Scale bar: 50 μm. (S) Representative image of TUNEL staining (green) in ischemic penumbra area, nucleus with DAPI (blue). GF (MCAO + MnPB) group treatment increased the number of TUNEL-positive cells. Scale bar: 100 μm. (T) Quantitative analysis of TUNEL positive cells (*n* = 3). Data are expressed as mean ± SD. *P < 0.05, ***P < 0.001, *vs*. Control group; #*P* < 0.05, *vs*. MCAO group (J, L–N: one-way analysis of variance followed by Tukey’s *post hoc* test; O, Q, T: unpaired *t*-test). CV: Coefficient of variation; DAPI: 4′,6-diamidino-2-phenylindole; GF: germ-free; HE: hematoxylin and eosin; LDA: linear discriminant analysis; MCAO: middle cerebral artery occlusion; MnPB: manganese-iron Prussian blue; SCFA: short-chain fatty acid; TUNEL: terminal deoxynucleotidyl transferase dUTP nick-end labeling; WT: wild type.

The effect of MnPB nanozymes on the function of gut microbiota, especially in relation to SCFA production, was a key area of exploration in this study. PICRUSt2 software was used to compare the predicted sequences with existing phylogenetic trees for species annotation, and Integrated Microbial Genomes microbial genome data were used to infer the functional gene composition of the samples by analyzing the species composition information obtained from 16S ribosomal RNA sequencing data. The shifts in the gut microbiota post-MnPB nanozyme treatment were further explored for their functional implications on the macrogenome, using PiCRUSt to predict metabolic pathways. Although no significant differences were observed in the overall metabolic pathway enrichment among the groups (**Figure 4A**), the MCAO group exhibited a higher relative abundance of genes associated with metabolic diseases (**Figure 4B**). Specifically, genes linked to carbohydrate metabolism and membrane transport were notably reduced in the MnPB group compared with the MCAO group (**Figure 4C**). Furthermore, there was a significant decrease in genes related to Microbial metabolism in diverse environments and ABC transporters in the MnPB group, while genes associated with biosynthesis of amino acids, potentially involved in SCFA production, were increased (**Figure 4D**). To identify bacterial taxonomic markers associated with MCAO, LEFSe analysis was conducted (**Figures 3J** and **4E**). The MCAO group showed a significant increase in Bacteroidia and Proteobacteria and a decrease in *Firmicutes* and *Bacilli* compared with the normal group. These changes suggest that MnPB nanozymes may improve infarct symptoms and neurobehavioral alterations by reducing gut microbiota dysbiosis. At the family level, there was a significant increase in the beneficial *Lactobacillaceae* (**Figure 4F**) and a decrease in the conditionally pathogenic *Enterococcaceae* (**Figure 4G**). The literature suggests that short-chain fatty acid producers, such as *Clostridia* and *Ruminococcaceae*, can mitigate neurological deficits and inflammation post-stroke (Zeng et al., 2019), enhancing SCFA concentrations in IS mice (**Figure 4H** and **I**). This finding indicates that MnPB nanozymes might ameliorate IS by modulating host metabolic pathways, such as the butyrate metabolism pathway, which is linked to inflammatory suppression and gut homeostasis maintenance (Patnala et al., 2017). To explore the mechanism by which MnPB nanozymes improve IS symptoms, SCFA levels in the feces and brain tissue were measured. A significant difference in SCFA production among the Control, MCAO, and MnPB groups was observed in the feces (*P* < 0.05; **Figure 4J**). **Figure 4K** shows the CV value of the mixed solution of brain tissue for quality control assessment, indicating the stability and reliability of the data. Additionally, the content of specific SCFAs in the brain tissue—namely butyric acid, 2-methylbutyric acid, and valeric acid—was significantly lower in the MCAO group and increased with MnPB treatment (**Figure 4L–N**). It has been reported that butyric acid, in particular, is effective in restoring gut microbiota balance, repairing microbiota leakage, reducing cerebral edema, and lowering lipid levels, and is thus a potential therapeutic option for IS (Rekha et al., 2024). Furthermore, NaB acts as a neuromodulator within the central nervous system, enhancing phosphoinositide 3-kinase (PI3K) expression, promoting Akt phosphorylation, reducing neuronal apoptosis, and decreasing infarct size and cerebral edema by activating the G protein-coupled receptor 41 (GPR41) receptor (Zhou et al., 2021). Analysis of SCFA concentrations in the gut contents of rats with post-stroke cognitive impairment revealed associations between SCFA levels and the occurrence and prognosis of post-stroke cognitive deficits (Chen et al., 2019c).

To test whether blocking SCFA signaling could eliminate the effect of MnPB nanozymes in the MCAO model, we administered MnPB nanozymes to WT and GF mice after MCAO. Once the treatment period was complete, neurobehavioral assessments were conducted, complemented by TTC staining of brain tissue and histological examination with HE staining. On the eighth day of treatment, behavioral patterns across all groups were evaluated using the Zea-Longa scoring system. As shown in **Figure 4O**, the score for mice in the WT (MCAO + MnPB) group was 1.5 ± 0.55, while the score for the GF mice was 2.67 ± 0.52. These results indicate that germ-free mice recovered far less neurological function than WT mice. To visually assess the therapeutic potency of MnPB nanozymes, TTC-stained brain sections from both groups of MCAO mice were examined on day 8 (**Figure 4P**). A significant increase in cerebral infarct volume from 20.03% ± 1.74% to 33.50% ± 3.49% was observed after treatment in the WT (MCAO + MnPB) group (**Figure 4Q**), showing that the MnPB nanozymes were less effective in GF mice than in WT mice. Reduced cell volume and marked nuclear atrophy were observed in the ischemic penumbra region in the GF (MCAO + MnPB) group relative to the WT (MCAO + MnPB) group (**Figure 4R**). Next, TUNEL staining was used to observe the effect of MnPB nanozymes on cell apoptosis in the ischemic penumbra area in the two groups (**Figure 4S**). Fewer apoptotic cells were observed in the WT (MCAO + MnPB) group, while the number of TUNEL-positive cells was significantly increased in the GF (MCAO + MnPB) group (**Figure 4T**). These findings confirmed that MnPB nanozyme treatment significantly improved IS symptoms and promoted brain recovery in WT mice subjected to MCAO but had no effect in GF mice lacking microflora. The concentration of total SCFAs in the intestine of GF mice is about 1/100 that of conventional mice (Høverstad and Midtvedt, 1986), and thus using this model is equivalent to inhibiting SCFA production; and indeed, no therapeutic effect of MnPB on IS was observed in these mice. These results demonstrated that MnPB nanozymes could enhance SCFA content by modulating the gut microbiota, which might be an important mechanism underlying their beneficial effects on ischemic stroke.

### MnPB nanozymes exert anti-inflammatory effects in middle cerebral artery occlusion mice

**Figure 5:**
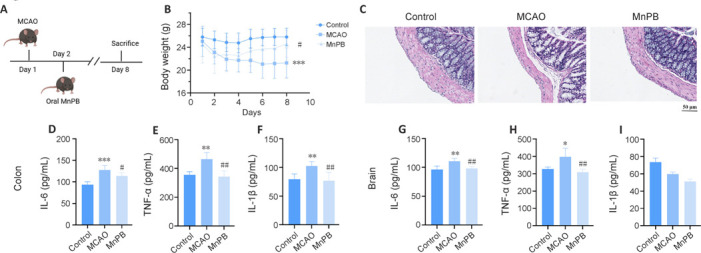
MnPB nanozymes demonstrate superior therapeutic efficacy in the MCAO mouse model. (A) Schematic representation of the experimental design, illustrating the induction of the MCAO model in mice and the subsequent oral administration of treatments commencing from the second day post-induction, with all mice being sacrificed on the 8^th^ day for further analysis. Created with BioRender.com. (B) Graphical depiction of body weight changes in mice throughout the experimental period, highlighting the differential effects of the MCAO model and MnPB nanozyme treatment (*n* = 8). (C) A series of representative images of hematoxylin and eosin staining on colon tissue from each group, revealing the histological effects of the MCAO model and the ameliorative impact of MnPB nanozymes (*n* = 3). Scale bar: 50 μm. (D, G) Quantitative analysis of IL-6 levels in both colon (D) and brain tissues (G), showing an increase in the MCAO group that was reversed by MnPB nanozyme treatment (*n* = 5). (E, H) Assessment of TNF-α levels in colon and brain tissues, indicating a significant upregulation in the MCAO group, with MnPB nanozymes effectively attenuating the rise of TNF-α in the colon. (F, I) Examination of IL-1β levels in colon tissue, where the MCAO group exhibited an increase that was mitigated by MnPB administration. Additionally, the changes in IL-1β content in brain tissue are presented (*n* = 5). Data are expressed as mean ± SD. *P < 0.05, **P < 0.01, ***P < 0.001, *vs*. Control group; #*P* < 0.05, ##*P* < 0.01, *vs*. MCAO group (one-way analysis of variance followed by Tukey’s *post hoc* test). IL-1β: Interleukin-1β; IL-6: interleukin-6; MCAO: middle cerebral artery occlusion; MnPB: manganese-iron Prussian blue; TNF-α: tumor necrosis factor α.

Inflammation is a major contributor to the pathophysiology of ischemic stroke. Therefore, evaluating the anti-inflammatory effects of MnPB nanozymes in a mouse model of MCAO mice is of great significance. As shown in **Figure 5A**, 2 days after mice were subjected to MCAO, they were treated with NS or MnPB nanozymes, and the effects of treatment on the physiological and inflammatory responses in the mice were observed. Notably, the body weight of the MCAO mice decreased more significantly than those treated with MnPB nanozymes, as depicted in **Figure 5B**. SCFAs, metabolites produced by the gut microbiota, energize colonic mucosal cells, stimulate cell metabolism and growth, and regulate the functions of intestinal epithelial and immune cells, thus preserving the integrity of the intestinal mucosal barrier (Parada Venegas et al., 2019). Our findings indicate that MnPB nanozymes increased SCFA levels, which in turn influenced colonic mucosal homeostasis. HE staining of colonic tissues, as shown in **Figure 5C**, revealed increased edema, mild inflammation, and notable damage to epithelial cells or mucin depletion in the MCAO group. By contrast, treatment with MnPB nanozymes effectively mitigated the morphological damage to colonic tissues induced by MCAO. Lipopolysaccharide, a component of Gram-negative bacteria, can lead to an overabundance of inflammatory mediators, exacerbating tissue and organ damage (Dong et al., 2014).

Next we used enzyme-linked immunosorbent assay kits to measure the levels of pro-inflammatory cytokines, including IL-1β, IL-6, and TNF-α, in the colon and brain tissues. The results, as illustrated in **Figure 5D–F**, demonstrated significantly elevated expression levels of these cytokines in the MCAO group than in the Control group. Interestingly, the brain tissue cytokine levels mirrored those found in the colon, with the exception of IL-1β, which did not follow the same trend, as shown in **Figure 5G–I**. In summary, the data suggest that the therapeutic efficacy of MnPB nanozymes in MCAO mice may be attributed to their regulatory effects on pro-inflammatory cytokines, potentially reducing inflammation and promoting tissue recovery.

### MnPB nanozymes mitigate oxidative stress and apoptosis via the TLR4/NF-κB signaling pathway

**Figure 6:**
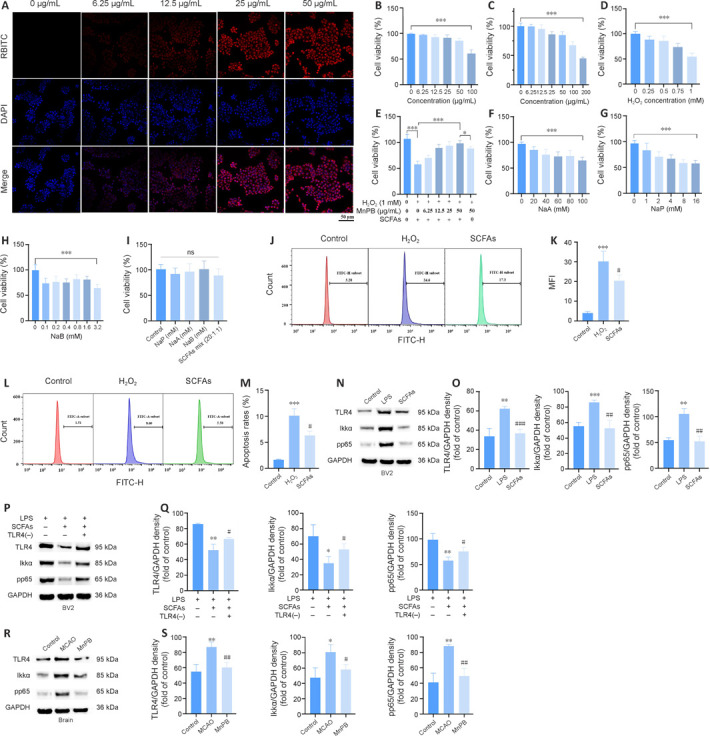
Therapeutic mechanisms of MnPB nanozymes and SCFAs in MCAO model. (A) Illustration of the concentration-dependent cellular uptake of MnPB nanozymes, highlighting the biocompatibility and internalization efficiency in NCM460 cells. Scale bar: 50 μm. (B) Assessment of the toxic effects of MnPB nanozymes on NCM460 cells, demonstrating safety within a concentration range of 0–50 μg/mL (*n* = 5). (C) Assessment of the toxic effects of MnPB nanozymes on BV-2 cells, demonstrating safety within a concentration range of 0–50 μg/mL (*n* = 5) (D) Examination of the toxic impact of H_2_O_2_ on BV-2 cells, establishing a baseline for oxidative stress-induced injury (*n* = 5). (E) Evaluation of the protective effects of different concentrations of MnPB nanozymes and SCFAs on cell survival in an *in vitro* oxidative stress injury model induced by H_2_O_2_ (*n* = 5). (F–H) Determination of the cytotoxicity of different concentrations of sodium acetate (NaA), sodium propionate (NaP), and sodium butyrate (NaB) on BV-2 cells after a 12-hour treatment period using the CCK-8 kit (*n* = 5). (I) Assessment of the combined toxicity of NaA, NaP, NaB, and SCFAs mixtures on BV-2 cells after a 12-hour exposure, confirming the safety of the selected concentration ratios (*n* = 5). (J) Utilization of the DCFH-DA assay to measure the reactive oxygen species (ROS) scavenging effect of varying concentrations of SCFAs. (K) Quantitative analysis of ROS spectral intensity, providing insights into the antioxidant capacity of SCFAs (*n* = 3). (L) Flow cytometry results depicting the apoptotic effects in BV-2 cells under oxidative stress. (M) Statistical analysis of apoptosis rates, revealing the protective role of SCFAs against cell death (*n* = 3). (N) Analysis of LPS-induced inflammation in BV-2 cells and the subsequent impact of SCFAs supplementation on the expression of TLR4, IKKα, and p65, as detected by western blotting. (O) Quantitative analysis of protein expression levels, indicating the modulatory effects of SCFAs on the TLR4/NF-κB pathway (*n* = 3). (P) Western blot bands of TLR4, IKKα, and pp65 expression in BV-2 cells following blockade of the TLR4 pathway, further elucidating the mechanism of action (*n* = 3). (Q) Quantitative analysis of protein expression levels, reinforcing the role of TLR4/NF-κB signaling in the observed effects (*n* = 3). (R) Western blot bands of TLR4, IKKα, and pp65 expression in brain tissues, providing *in vivo* evidence of the pathway’s involvement. (S) Quantitative analysis of protein expression density, underscoring the significance of TLR4/NF-κB modulation in the therapeutic effects (*n* = 3). Data are expressed as mean ± SD. *P < 0.05, **P < 0.01, ***P < 0.001, *vs.* Control group; #*P* < 0.05, ##*P* < 0.01, ###*P* < 0.01, *vs*. MCAO group (one-way analysis of variance followed by Tukey’s *post hoc* test). CCK-8: Cell counting kit-8; GAPDH: glyceraldehyde 3-phosphate dehydrogenase; IKKα: inhibitory kappa B kinase α; MCAO: middle cerebral artery occlusion; MnPB: manganese-iron Prussian blue; NF-κB: nuclear factor κB; RBITC: Rhodamine B isothiocyanate; SCFA: short-chain fatty acid; TLR4: Toll-like receptor 4.

Understanding the molecular mechanisms by which MnPB nanozymes exert their protective effects, especially regarding oxidative stress and apoptosis, is crucial for elucidating their therapeutic potential. To elucidate MnPB cellular uptake and biocompatibility, we labeled MnPB nanozymes with RBITC, co-incubated them with NCM460 cells for 4 hours, and observed cellular uptake under a fluorescence microscope. As **Figure 6A** illustrates, after 4 hours of co-incubation, NCM460 nuclei were uniformly stained blue by 4′,6-diamidino-2-phenylindole, and the cytoplasm showed red fluorescence due to RBITC-MnPB uptake. The merge plot indicates blue nuclear fluorescence and red cytoplasmic regions within the same cells, signifying effective cellular uptake of RBITC-MnPB by NCM460 cells within 4 hours, with fluorescence intensity increasing in a concentration-dependent manner. We assessed the cytotoxic effects of varying concentrations of MnPB nanozymes (0–100 μg/mL) on colonic epithelial cells (NCM460) using the CCK-8 kit. As depicted in **Figure 6B**, NCM460 cells exhibited no significant cytotoxicity when exposed to concentrations of MnPB up to 50 μg/mL. As **Figure 6C** shows, MnPB nanozymes were safe for BV-2 cells within the 0–50 μg/mL range. To explore the impact of MnPB nanozymes on H_2_O_2_-induced oxidative stress in BV-2 cells, we assessed cell viability using a CCK-8 kit. **Figure 6D** shows that 1 mM H_2_O_2_ reduced cell survival. However, preincubation of cells with different concentrations of MnPB nanozymes and SCFAs for 4 hours, followed by replacement of the medium with fresh medium containing 1 mM H_2_O_2_, resulted in a significant increase in cell viability that increased with increasing concentrations of MnPB nanozymes. At an MnPB concentration of 50 μg/mL, the absence of SCFAs resulted in a slight decrease in cell viability (**Figure 6E**). This is an important reason why we did not supplement SCFAs alone, but rather regulate the increase of SCFAs through MnPB nanozymes to alleviate oxidative stress and reduce inflammation. This phenomenon is consistent with the results from the animal experiments: although MnPB itself reduced oxidative stress, MnPB nanozyme–mediated regulation of SCFAs increased this effect. SCFAs, primarily acetate, propionate, and butyrate, are produced by gut microbiota during carbohydrate fermentation and modulate various host brain physiological functions. Recent studies have shown a strong association between microglial maturation and brain SCFAs (Caetano-Silva et al., 2022). SCFAs regulate inflammation, glucose metabolism, and blood pressure, and affect blood–brain barrier stability and microglial maturation/activation (Caetano-Silva et al., 2022). We determined the toxicity of different SCFA concentrations on BV-2 cells using the CCK-8 assay. **Figure 6F–H** shows the safe concentration ranges for NaA, propionate, and butyrate. Considering the typical 1:1 ratio of propionate to butyrate in organisms, we chose a mixture of 20 mM NaA, 1 mM NaB, and 1 mM NaP, which did not affect cell viability after treatment for 12 hours (**Figure 6I**). While MnPB nanozymes are known to alleviate oxidative stress, the role of MnPB nanozymes in regulating oxidative stress in the context of increased SCFAs concentrations is still unknown. We next supplemented BV-2 cells with SCFAs and assessed apoptosis and changes in ROS content. To assess the effect of ROS scavenging on H_2_O_2_-induced oxidative stress, we used the 2,7-dichlorofluorescein diacetate live cell probe (**Figure 6J**). Flow cytometry results indicate a significant reduction in ROS production in cells exposed to SCFAs compared with control cells (**Figure 6K**). Additionally, flow cytometry was used to assess H_2_O_2_-induced apoptosis (**Figure 6L**), which was effectively decreased by supplementation with SCFAs (**Figure 6M**). To further investigate the mechanism by which the SCFAs inhibited LPS-induced inflammation in BV-2 cells, we examined the expression of key molecules in the TLR4 inflammatory pathway––TLR4, IKKα, and pp65––by western blot (**Figure 6N** and **O**). These results suggest that the SCFA mixture may inhibit inflammation via the TLR4/NF-κB pathway. Building on this, we explored the potential mechanism of MnPB by blocking the TLR4/NF-κB pathway (**Figure 6P** and **Q**). Treatment with a TLR4 inhibitor reversed the therapeutic effect of SCFAs on inflammation, and this effect was corroborated by *in vivo* protein expression levels in brain tissue (**Figure 6R** and **S**). Compared with the normal group, the MCAO group showed significantly increased levels of TLR4, IKKα, and pp65 in brain tissue (*P* < 0.01, *P* < 0.05, or *P* < 0.01), indicating involvement of the TLR4/NF-κB pathway in MnPB’s therapeutic effects. The results suggest that MnPB nanozymes could mitigate oxidative stress and apoptosis through modulating the TLR4/NF-κB signaling pathway, providing insights into their underlying therapeutic mechanisms.

## Discussion

IS triggers a cascade of complex pathophysiological reactions. Notably, IS onset and reperfusion can lead to a significant surge in the production of ROS, including hydrogen peroxide, hydroxyl radicals, and superoxide anions (Zhang et al., 2019; Wu et al., 2025). These ROS can induce extensive neuronal cell death, exacerbating brain tissue damage (Zhao et al., 2018a). ROS serves as a critical signaling molecule, initiating a cascade of events that includes the activation of microglia, the recruitment of peripheral white blood cells, and the secretion of cytokines by inflammatory cells, all of which contribute to the inflammatory response (Mittal et al., 2014). To counteract this, Prussian blue nanoparticles have emerged as a promising strategy for their ability to scavenge ROS, which is attributed to their intrinsic properties that mimic the functions of enzymes such as catalase and superoxide dismutase (Zhou et al., 2019). Building upon this foundation, we developed a novel class of nanozymes, MnPB, which exhibit enhanced ROS-scavenging capabilities due to their improved enzyme-like activities and porous structure. These MnPB nanozymes not only demonstrate superior biocompatibility but also possess a robust capacity for ROS neutralization, positioning them as a potential therapeutic agent for various oxidative stress-related conditions (Hu et al., 2024). Here we show that these MnPB nanozymes are also able to modulate the gut microbiota. By adjusting the composition and metabolic activities of the gut microbiota, these nanoparticles can potentially influence the host’s response to various stimuli, including ischemic events (Koszewicz et al., 2021). This modulation of the gut microbiota by MnPB nanoparticles could provide a novel avenue for treating conditions like IS, where the gut-brain axis plays a pivotal role in disease progression and recovery. Our study presents the first evidence of the impact of MnPB nanozymes on the gut microbiota following IS. The results indicate that these nanozymes can significantly alter the gut microbiota’s composition, potentially leading to beneficial metabolic shifts that could support recovery. In our moused model of IS, treatment with MnPB nanozymes led to a notable reduction in neurological deficit scores and an improvement in various neurological functions, including motor coordination and cognitive abilities. Ischemic events can lead to neuronal damage in critical brain regions such as the hippocampus, which is integral to learning and memory processes (Sadeghzadeh et al., 2023). This damage could explain the cognitive impairments observed in the IS model. However, our findings suggest that MnPB nanoparticles can ameliorate these effects, preserving neuronal integrity and promoting functional recovery. In summary, our research highlights the multifaceted therapeutic potential of MnPB nanoparticles in the context of IS, emphasizing their ability to modulate the gut microbiota and ameliorate the associated neurological deficits.

A previous study demonstrated the intricate interplay between the gut microbiota and stroke, with significant variations observed in the microbial composition of stroke patients (Malkki, 2016). A more recent study indicated that specific genera such as Paramycetes, Oscillatoria, and Enterobacteriaceae are more prevalent in stroke patients, while Prevotella, Roseburia, and Enterococcus fecalis are more abundant in healthy individuals (Peh et al., 2022). In the context of IS, gut dysbiosis is closely linked to metabolic alterations and inflammatory responses. Moreover, certain gut microbial genes and their associated metabolites have emerged as potential biomarkers for stroke risk prediction and prognostic assessment (Li et al., 2019; Zheng et al., 2022). Paralleling these clinical findings, animal models of stroke have exhibited similar shifts in gut microbiota composition. For instance, overgrowth of *Anaplasma* and *Aspergillus* was noted in mice subjected to MCAO (Singh et al., 2016). Post-stroke rats have shown an increase in the abundance of opportunistic pathogens, including *Alistipes*, *Mycobacterium anisopliae*, *Klebsiella*, *Shuttleworthia*, *Haemophilus*, *Clostridium*, *Enterococcus fecalis*, *Aspergillus*, and *Bacteroidetes* (Chen et al., 2019a). In our study, we subjected mice to MCAO, treated them with MnPB nanozymes, and conducted an analysis of the gut microbiota to explore its role in these mice. We discovered that MnPB nanozymes can modify the alpha diversity and overall structure of the gut microbiota in the aftermath of stroke. More detailed examination of the microbiota’s structural composition revealed that MnPB nanozymes significantly enhanced the presence of bacteria potentially involved in SCFA production.

*Lachnospiraceae* and *Clostridia* are the predominant bacterial groups responsible for SCFA production (Chen et al., 2019b). SCFAs are pivotal gut microbial metabolites in mammals, serving as essential substrates for cholesterol, glucose, and lipid metabolism, and contributing nearly 10% to daily caloric needs (LeBlanc et al., 2017). Beyond their metabolic roles, SCFAs also exert anti-inflammatory effects by modulating T cells through activation of G protein-coupled receptors (Al Nabhani et al., 2019). Additionally, SCFAs contribute to protection and repair of the gut mucosal barrier by stimulating mucus secretion and enhancing the expression of tight junction proteins (Zhao et al., 2022). Lactobacillus abundance has been noted in patients with cerebral infarction, suggesting a potential beneficial role in stroke recovery (Li et al., 2020). In our study, we observed a significantly higher abundance of lactobacilli in the feces of the MnPB nanozyme-treated group compared with the MCAO group. This finding is particularly intriguing because Bourriaud and colleagues have shown that butyrate-producing bacteria can ferment lactic acid to produce butyrate, thereby reducing the inflammatory response and protecting the brain from injury (Bourriaud et al., 2005). Given that MnPB nanozymes elevate the abundance of bacteria potentially capable of producing SCFAs, the observed increase in Lactobacillus could lead to enhanced lactic acid fermentation into butyrate. This increase in butyrate production may, in turn, mitigate neuroinflammation during stroke, offering a novel perspective on the neuroprotective mechanisms of MnPB nanozymes.

Dysregulation of the gut microbiota can have profound effects on gut barrier integrity and systemic inflammation because it is directly linked to elevated levels of inflammatory biomarkers, including TLR4. TLR4 upregulation has been implicated in a range of conditions such as depression, insulin resistance, and inflammation (Dapito et al., 2012; Kim et al., 2012). In our study, treatment with MnPB nanozymes significantly lowered the concentrations of TNF-α, IL-1β, and IL-6 in both the colon and brain tissues of MCAO-induced mice compared with the control group. This reduction may be due to increased expression of TLR4 and NF-κB, as well as the compromised gut barrier. The TLR4/TNF-α signaling pathway is thought to be the principal mechanism coupling the gut microbiota with brain function (Liu et al., 2018), with TLR4 signaling being intricately connected to gut homeostasis, permeability, and inflammatory responses. Our findings indicate that MnPB nanozymes effectively inhibited the MCAO-induced increase in the expression of key proteins within the TLR4/NF-κB signaling pathway, modulating the gut microbiota and ameliorating the pathological effects of IS. The elevation of SCFAs triggered by MnPB nanozymes was closely associated with inhibition of the TLR4/NF-κB signaling pathway. However, our study had some limitations, in that we were not been able to explore this mechanism in depth. We did not thoroughly investigate the relevant mechanisms and pharmacokinetics of Prussian blue nanomaterials. Instead, our focus was primarily on the distribution of drugs within various organs. We intend to pursue further research in this area in the future. Additionally, in this study, we conducted a preliminary screening of specific SCFAs-producing bacteria. Future research and experiments related to fecal microbiota transplantation will be undertaken to expand upon these findings. In preclinical studies, the presence of sex hormones, particularly estrogen in female mice, is known to exert neuroprotective effects during ischemia-reperfusion injury. These hormones can significantly influence vascular responses, inflammatory pathways, and neuroprotective mechanisms. Furthermore, the hormonal fluctuations that occur throughout the estrous cycle in female mice introduce variability that can compromise the stability and reliability of experimental outcomes. To enhance the reproducibility and comparability of our data, we opted to use only male mice in our experiments. This strategy was designed to minimize the confounding variables associated with sex, thereby ensuring a more consistent and robust experimental framework. Furthermore, the levels of TNF-α, IL-1β, and IL-6 induced by MCAO were decreased in mice treated with MnPB nanozymes, suggesting that SCFAs produced through modulation of the gut microbiota by MnPB nanozymes contribute positively to therapeutic outcomes in mice subjected to MCAO. In summary, our study demonstrates that MnPB nanozymes ameliorate neurological damage in mice subjected to MCAO by preserving the integrity of the gut barrier, curbing TLR4/NF-κB signaling, and regulating the gut microbiota and SCFA production. These findings pave the way for future investigations into the role of MnPB nanozymes within the framework of the gut-brain axis.

## Additional files:

***Additional Figure 1:*** *Flowchart of the animal experiments.*

Additional Figure 1 Flowchart of the animal experiments.
16S rRNA: 16S ribosomal RNA; GF: germ-free; HE: hematoxylin and eosin; IL-1β: interleukin-1β; IL-6: interleukin-6; MCAO: middle cerebral artery occlusion; MnPB: manganese-iron Prussian blue; SCFA: short-chain fatty acid; TNF-α: tumor necrosis factor α; TTC: 2,3,5-triphenyltetrazolium chloride; TUNEL: terminal deoxynucleotidyl transferase dUTP nick-end labeling; WT: wild type.

***Additional Table 1:*** *Number of experimental animals in the in vivo experiments.*

## Data Availability

*All data relevant to the study are included in the article or uploaded as Additional files.*
